# An audit of registered radiology equipment resources in Uganda

**DOI:** 10.11604/pamj.2020.37.295.22046

**Published:** 2020-12-02

**Authors:** Elsie Kiguli-Malwadde, Rosemary Byanyima, Michael Grace Kawooya, Aloysius Gonzaga Mubuuke, Roy Clark Basiimwa, Richard Pitcher

**Affiliations:** 1 Health Workforce Education and Development, African Centre for Global Health and Social Development, Plo13B, Acacia Avenue, Kampala, Uganda,; 2 Department of Radiology, School of Medicine, College of Health Sciences, Makerere University, Kampala, Uganda,; 3 Department of Radiology, School of Medicine, Mulago Hospital, Kampala, Uganda,; 4 Ernest Cook Ultrasound Research and Education Institute (ECUREI), Mengo Hospital, Kampala, Uganda,; 5 Joint Clinical Research Centre, Kampala, Uganda,; 6 Division of Radiodiagnosis, Department of Medical Imaging and Clinical Oncology, Faculty of Medicine and Health Sciences, Stellenbosch University, Stellenbosch, South Africa

**Keywords:** Audit, radiology, imaging, equipment, Uganda

## Abstract

**Introduction:**

the third Sustainable Development Goal (SDG) relates to Universal Health Coverage (UHC) and provision of quality essential health services. The Government of Uganda has operationalized this through the National Health Policy which stresses the importance of availability of functioning medical equipment in health facilities. There have been efforts by the Ministry of Health and Atomic Energy Council in Uganda to compile an inventory of imaging equipment in the country, however, this information has not been widely published. The purpose of this study was to conduct an audit of registered radiology equipment in Uganda and establish their functional status.

**Methods:**

a cross-sectional descriptive study that involved a desktop review of the equipment registry at the Uganda Atomic Energy Council was conducted. Data was collected on a number of variables including type of equipment, location, functional status, modality and density per million people.

**Results:**

the audit revealed 625 pieces of equipment spread over 354 health facilities. The majority (397) were plain X-ray machines followed by dental X-ray machines at 120. There were only 3 Radiotherapy machines. Most were recorded as being functional with only 0.1% of the equipment non-functional. Most of the equipment was in the central region which has the third highest population density. The majority of the equipment belonged to private health facilities.

**Conclusion:**

Uganda lags behind the WHO recommended ratio of equipment versus the population (20 per million population). Most of the equipment is the plain X-ray machine with a few more advanced technologies in both public and private health facilities.

## Introduction

The United Nations (UN) 2030 Agenda for Sustainable Development calls for unified global action to address the economic, social and environmental priorities reflected in the 17 Sustainable Development Goals (SDGs) [1]. Healthcare is particularly addressed in the third SDG (SDG3). This has 13 targets, covering all major health imperatives, including Universal Health Coverage (UHC). The vision of UHC is to provide all people with quality essential health services. The Government of Uganda, in its policy document, the Health Sector Development Plan (HSDP) 2015-2020, identifies availability and functionality of medical equipment as one of the critical requirements for delivery of quality healthcare services [2]. Therefore it is important for the Ugandan government to have statistics and data concerning the status of radiology equipment in Uganda. This will assist in proper planning of healthcare delivery. In recent years, public-private partnerships have been initiated by the government to acquire and develop nationwide infrastructure as well as offer opportunities to improve service delivery. This thus demonstrates that the government of Uganda recognizes the role of the private health sector in the delivery of quality health services to its population [3].

**Figure 1:**
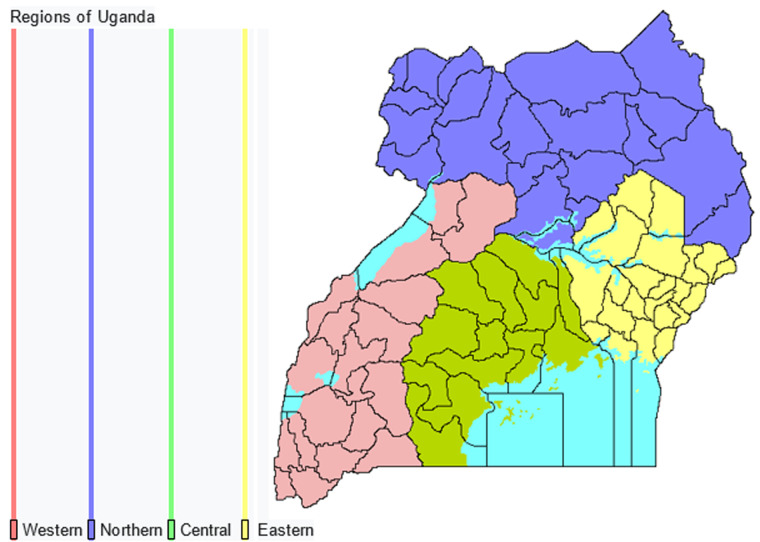
map of Uganda by region

Uganda is a land-locked country with a total land mass of 241,559 square kilometers, including approximately 37,000 square kilometers of water coverage. The most recent National Population Census (2014) showed that the country's population was at 34.8 million people, with a rural predominance (28.4 million people, 82%). This reflects a substantial population growth from that recorded in the previous census. With an annual growth rate of 3%, Uganda's population is projected to soar in the future. Indeed, in 2019, the Ugandan Bureau of Statistics estimated the Ugandan population to have increased to approximately 40.3 million people. The country has four major regions, namely Central, Western, Eastern and Northern (Figure 1). The population is distributed among the 4 regions as follows; Central - 11,184,500, Eastern - 10,512,200, Northern - 8,346,600 and Western - 10,264,700 with the estimated total population as 40,308,000. The total population in each region varies though not widely. However the northern region is vast thus less densely populated [4].

Developing countries account for 84 percent of global population, 90 percent of the global disease burden, and 20 percent of global Gross Domestic Product GDP, but only 12 percent of global health spending. At the same time, Low-Income Countries (LICs) are struggling with a large burden of communicable diseases, while also confronting increases in the prevalence of non-communicable diseases and injuries, a trend that will likely continue for some time. The availability of resources to meet these numerous health needs is limited [5]. Despite sustained economic growth and impressive income poverty reduction in Uganda, Uganda's Nominal GDP Per Capita was forecasted to be 770.062 USD in December 2019 as reported by International Monetary Fund - World Economic Outlook [6]. This still keeps Uganda in the low Income bracket as described by the World Bank. Despite the growth in Uganda's GDP between 2007 and 2016, the public health sector has not been able to attract adequate resources. The government's health budget has been on the decline as a proportion to both GDP and of general government expenditure. In monetary terms and according to the WHO GHED, total health expenditure per capita has fallen, after peaking at USD 63 per capita in 2010, to USD 38 per capita in 2016 [7]. Out of Pocket Expenditure (OOP) on health in Uganda stands at 40% as a percentage of total health expenditure which is an indicator of the adverse level of vulnerability to low income households given current levels of morbidity [8]. Uganda currently has only 5% of the population covered under health insurance and only 11% of persons aged over 15 years are even aware of health insurance [4,8]. This shows the challenges faced by the Uganda government in meeting the health needs of its population.

According to Uganda Health Sector Development Plan 2015/16-2019/20 (HSDP), health impact indicators that track the progress towards UHC are the Maternal Mortality Rate (MMR) at 368, Neonatal Mortality Rate (NMR) at 27, Infant Mortality Rate (IMR) at 43, Under-Five Mortality Rate (U5MR) at 64, Total Fertility Rate (TFR) at 5.8 and Adolescent Pregnancy Rate (APR) at 25%. According to the mid-term review of the HSDP, even if some targets are met, there is still a lot that needs to be done, especially as the country approaches the end of the HSDP's timeline and the SDGs in the long-term [9].Tuberculosis (TB) is another highly prevalent and expensive disease in low-income countries including Uganda, even though most of the cost for the treatment is borne by external donors. The TB prevalence in Uganda is high at 253 TB cases per 100,000 population compared to 159 TB cases per 100,000 population reported in the 2015 WHO Global TB report [10]. For any TB patient to start treatment an X-ray of the chest has to be done. This highlights the great need for diagnostic imaging.

Healthcare technology, including diagnostic imaging, is acknowledged as an essential component of any healthcare system. Basic medical diagnostic imaging services, such as plain X-rays and ultrasound, are required for effective primary care of patients [2,11]. The current Uganda National Medical Equipment Policy (2009) recommends ultrasound and general radiography equipment at Health Centre IV. Government health services in Uganda are structured into national and regional referral public hospitals and general hospitals. At district level; it is divided into four levels (I-IV). Health Centre Level I (HC-I) is the lowest level in the health system, and comprise a Village Health Teams (VHTs) or individual health volunteer (that may or may not be formally trained) serving to link the community to the National Health Service. Health Centre IIs (HC-II), also known as dispensaries, these are parish level facilities that serve roughly 5000 people. They are led by an enrolled nurse who works with a midwife, and two nursing assistants.

Health Centre III (HC-III) facilities serve a sub-county of approximately 20,000 people, and supervise community health workers and the HC-IIs within their jurisdiction. Health Centre level IV (HC-IV)/District Hospitals serve a county (approximately 100,000 people) and offer the highest level of service to the district. They offer inpatient care. Above this are the regional referral and one national referral hospital [12]. Access to these basic imaging modalities should be seen as integral to achieving UHC. The World Health Organization (WHO) has postulated that 90% of all imaging requirements in Low- and Middle-income countries (LMICs) can be met by the provision of one X-ray unit and a single ultrasound machine for every 50,000 people, or 20 units per million people [1,13]. This figure may serve as the yardstick for evaluating access to basic imaging at country-level. Robust country-level data are thus required to assess the extent to which countries meet basic imaging resource targets. In May 2007, the 60^th^ UN World Health Assembly adopted Resolution 60.29, urging member states to “collect, verify, update and exchange information on health technologies, in particular medical devices, as an aid to their prioritization of needs and allocation of resources”[13] .

There is little published work on in-country imaging resources globally, worse still in Uganda. One survey quantified the imaging needs in five selected hospitals in Uganda, based on the disease burdens in these hospitals and reported on how the existing imaging equipment addressed these needs, concluding that existing imaging equipment met only 36% of the imaging needs in these hospitals. This survey also reported that up to 50% of patients attending these selected hospitals required some type of imaging, but mostly ultrasound and X-ray [14,15]. For Uganda, the Atomic Energy Council (AEC) maintains an up to date countrywide inventory of radiology imaging devices. The Ministry of Health Infrastructure Department with assistance from the Infectious Disease Institute, Makerere University (IDI) has recently embarked on expanding this Medical Equipment Database, to include year of manufacture and functional status. This information is geared to inform health planning in the country, but is not currently available. The drivers and determinants of these resources remain poorly understood and the relationship between national healthcare expenditure, national health indicators and in-country access to diagnostic imaging has not been rigorously assessed. Three other similar studies have been done in South Africa, Tanzania and Zimbabwe [16-18].

A country's official national registry of diagnostic radiology equipment can assist in defining health coverage. Diagnostic imaging equipment that utilizes ionizing radiation is generally licensed for use in a specific location. Such locations have typically been formally evaluated and found to meet the infrastructural requirements for safe operation, such as adequate radiation shielding and appropriate electrical supply. Relocation of radiology equipment typically requires re-licensing and infrastructural development. Furthermore, imaging equipment utilizing ionizing radiation are only operated by a registered radiation worker. An inventory of licensed imaging equipment thus provides robust data on the number and distribution of units, as well as broader insights into the so-called “imaging enterprise” [19]. Nonetheless, there appears to be scant global recognition of the potential role of registered diagnostic imaging equipment in reflecting country-level health coverage. It is in this context that the African Centre for Global Health and Social Transformation (ACHEST), the Division of Radiodiagnosis in the Department of Medical Imaging and Clinical Oncology in the Faculty of Medicine and Health Sciences (FMHS) at Stellenbosch University (SU), Department of Radiology, Makerere University and the Uganda Atomic Energy Council conducted an audit of registered Ugandan diagnostic and therapeutic radiology equipment resources.

## Methods

A cross-sectional study was carried out in August 2019 in Uganda. The country's national registered diagnostic imaging equipment inventory, maintained by the AEC, was interrogated. Data on the number and location of general radiography, fluoroscopy, C-arm, interventional radiology, computed tomography (CT), mammography and dental radiography units were captured on a customized data sheet, with stratification by imaging modality, geographical region, and health-care sector (public/private). General radiography equipment was further stratified as fixed/mobile, digital/analogue and functionality at the time of last AEC inspection. Magnetic Resonance Imaging was excluded as it is not registered by The AEC. Results were presented as units per million people, by modality, geographical region and healthcare sector (public/private). Ugandan resources were compared with the WHO guidelines for basic imaging equipment, and with published equipment data of other African countries namely South Africa, Tanzania and Zimbabwe. Registered Ugandan diagnostic imaging resources were correlated with national economic indicators and the national SDG health target indicators.

## Results

The Ugandan population by region served by the radiology equipment is summarized in Table 1. There were 354 centers included in the data collected from AEC of Uganda with a total of 625 equipment units; the equipment modalities that were looked at included 397 plain radiography (PR) [divided into 199 Fixed PR (FPR) and 198 Mobile PR (MPR)], 120 Dental Radiography (DR), 28 C-Arm (CA), 25 Computed Tomography scan (CT), 20 Mammography (MM), 32 Fluoroscopy(FL) and 3 Radiotherapy (RT) [2 Co-60 Teletherapy and 1 Co-60 Brachytherapy HDR]. This means there are 15.5 pieces of equipment per million populations. Of all equipment in the database, less than 0.1% was not in current use. Plain radiography (PR): Overall, the largest number of equipment is PR accounting for 63.52% (397/625) of all the equipment. The private sector has 52.6% of the PR equipment. Most of them are in the central region 42.8%. Almost half of the PR equipment is serving 25% of the population. However, the density in the central region (15.2 per million populations) which is the highest for PR is still below the WHO recommended threshold. The rest of the regions are at half of the central region (Table 2, Table 3). Dental radiograph has an 8-fold discrepancy with other regions. Fluoroscopy: there is a 5-fold discrepancy for fluoroscopy in the central region compared to the rest of the region with 62.5% (20/32) of them in the private sector leaving only 37.5% (12/32) for the public sector. Most of the mammography equipment is in the central region at 75% (15/20) of which 60% (12/20) are in the private sector. The western region has only one mammography machine for a population of 10.26 Million, 25% of the Ugandan population. Computed tomography: out of 25 CTs, 18 (72%) are in private centers, 52% (13/25) are in the central region, while there are none in the northern region. There is a large disparity between the public and private (7:18). Other modalities: Positron Emission Tomography (PET-CT) and Digital Subtraction Angiography (DSA) are not available. Comparison with South Africa, Tanzania and Zimbabwe: Tanzania has the lowest density of equipment per population followed by Uganda, Zimbabwe and South Africa have densities higher than the recommended threshold by WHO, 34.8 and 26 per million population for PR (Table 4).

**Table 1:** Ugandan population by region

Region	Population (x 10^6^)	Area (Km^2^)	Population density (people/Km^2^)
Central	11.18	61,403.2	182
Eastern	10.51	39,478.8	266
Northern	8.35	85,391.7	98
Western	10.26	55,276.6	185
**Total**	40.3	241,550.3	166

**Table 2:** number of radiology equipment (units) by region, modality and health sector

		Central	Eastern	Northern	Western	Total
**FPR**	**Total**	86	39	32	42	199
	Gov't	21	14	11	12	58
	Private	65	25	21	30	141
**MPR**	**Total**	84	31	33	50	198
	Gov't	17	3	4	6	30
	Private	67	28	29	44	168
**DR**	**Total**	89	10	6	15	120
	Gov't	14	6	3	8	31
	Private	75	4	3	7	89
**CA**	**Total**	21	1	2	4	28
	Gov't	8	1	0	4	13
	Private	13	0	2	0	15
**CT**	**Total**	19	3	0	3	25
	Gov't	6	0	0	1	7
	Private	13	3	0	2	18
**MM**	**Total**	15	2	2	1	20
	Gov't	3	0	0	0	3
	Private	12	2	2	1	17
**FL**	**Total**	20	4	5	3	32
	Gov't	6	2	2	2	12
	Private	14	2	3	1	20
**RT**	**Total**	3	0	0	0	3
	Gov't	0	0	0	0	0
	Private	3	0	0	0	3

Fixed Plain Radiography (FPR), Mobile Plain Radiography (MPR), Dental Radiography (DR), C-Arm (CA), Computed Tomography (CT), MM Mammography, Fluoroscopy (FL) and Radiotherapy (RT)

**Table 3:** density of radiology equipment (units/106 people) by region, modality and health sector

		Central	Eastern	Northern	Western	Total
**FPR**	**Total**	**7.7**	**3.7**	**3.8**	**3.1**	**4.9**
	Gov't	1.8	1.3	1.3	1.2	1.4
	Private	5.8	2.4	2.5	2.9	3.5
**MPR**	**Total**	**7.5**	**3.0**	**4.0**	**4.8**	**4.9**
	Gov't	1.5	0.3	0.5	0.6	0.7
	Private	6.0	2.7	3.5	4.3	4.2
**DR**	**Total**	**7.9**	**1.0**	**0.7**	**1.5**	**3.0**
	Gov't	1.3	0.6	0.4	0.8	0.7
	Private	6.7	0.4	0.4	0.7	2.2
**CA**	**Total**	**1.9**	**0.1**	**0.2**	**0.4**	**0.7**
	Gov't	0.7	0.1	0	0.4	0.3
	Private	1.2	0	0.2	0	0.4
**CT**	**Total**	**1.7**	**0.3**	**0**	**0.3**	**0.6**
	Gov't	0.5	0	0	0.1	0.2
	Private	1.2	0.3	0	0.2	0.4
**MM**	**Total**	**1.3**	**0.2**	**0.2**	**0.1**	**0.5**
	Gov't	0.3	0	0	0	0.1
	Private	1.0	0.2	0.2	0.1	0.4
**FL**	**Total**	**1.8**	**0.4**	**0.6**	**0.3**	**0.8**
	Gov't	0.5	0.2	0.2	0.2	0.3
	Private	1.2	0.2	0.4	0.1	0.6
**RT**	**Total**	**0.3**	**0**	**0**	**0**	**0.1**
	Gov't	0	0	0	0	0
	Private	0.3	0	0	0	0.1

**Table 4:** comparison of Ugandan, Tanzanian, Zimbabwean and South African radiology equipment resources by modality

	Uganda	Tanzania	South Africa	Zimbabwe
**Demographics**				
Population (x10^2^ people)	40.3	59.7	59.3	13.0
Area (x10^3^ Km^2^)	241.6	890.1	1219.1	390.7
Population Density	166	67	48	33
GDP from World Bank in 2018 (Billion US$)	27.5	58	368.3	18
Health expenditure per capita in 2016 (US$)	37.6	35.5	428.2	94
**Total radiology equipment (in units per million population)**				
Plain radiography	9.6	9.0	34.8	26
Fluoroscopy	0.8	1.0	6.6	0.8
Mammography	0.5	0.8	5.0	0.8
CT	0.6	0.4	5.0	1.5

## Discussion

The purpose of this study was to conduct an audit of registered radiology equipment in Uganda. This is the first published quantitative analysis of registered diagnostic radiology equipment resources in Uganda. Conducting such an audit is important for planning purposes for the health care system if the number, type and functional status of the equipment is to be known. Findings from this audit will potentially be useful to the Ministry of Health and also provide a basis for such audits in other developing countries. In this study we discovered that there were 15.5 units of equipment per one million people, which is less than the WHO recommendation of 20 units per one million. It is also less than what has been reported from South Africa and Zimbabwe, but better than what has been reported from Tanzania. The good indicators reported from South Africa and Zimbabwe might possibly be due to the fact that those countries are putting in more resources to cater for radiology equipment for their population since their health expenditure per capita is more than that of Uganda. However, the fact that the Ugandan radiology equipment distribution per population is still below the recommended WHO number is a point of concern. If Uganda is to satisfactorily attain SDG3, the number of equipment per million population needs to increase through provision of the different radiology equipment across the country. This calls on the Ministry of Health to work together with other stakeholders to increase funding if Uganda is to attain the WHO recommended figure. It is well documented that this is linked to provision of efficient Primary Health Care which is important for achieving Universal Health Coverage (UHC) and also affects Uganda's ability to achieve the Sustainable Development Goals (SDGs). However, it is important to note that the WHO recommendation includes Ultrasound equipment which was not surveyed in this study. We also established that most of the radiology equipment in Uganda was in good functional status which is commendable bearing in mind that like most LICs, Uganda has very few Biomedical Engineers to keep and such equipment in good condition.

Regarding the equipment distribution across the country, this study showed a skewed distribution. Most of the radiology equipment was found within the Central region followed by the western region. This has been termed the capital city syndrome by Palmer *et al*. [19]. This is when most equipment is concentrated within cities, most specifically the capital city which in the case of Uganda is in the central region. This mal-distribution is not only for the equipment but also the radiology human resource since the availability of human resource depends on the availability of equipment. This is likely to lead to mal-distribution of service delivery where by only a small number of the people who need the services is reached. Significantly, the high end technology and most expensive equipment like the Computed Tomography (CT), Fluoroscopy (FL) and Radiotherapy (RT) equipment is in the central region. This could be influenced by the fact that most medical schools, tertiary hospitals and major referral hospitals and institutes are located within the central region. For example, Uganda has only one Cancer Institute which is in the Central region, so all the radiotherapy units are in the central region. At the same time the equipment is government owned. Notably there are plans to decentralize cancer care to all the regions and may change this mal-distribution in the near future [20]. One large privately owned faith based hospital is also planning to procure Radiotherapy (RT) equipment soon. The skewed distribution of radiology equipment therefore means that a significant number of people are likely not to get radiology services especially in cases when high-tech radiological investigations are needed. Therefore, the Ministry of Health needs to ensure that there is equitable distribution of equipment across all the regions in the country.

Equipment distribution is likely to be influenced by limited funding. It might therefore be difficult to have high-end equipment evenly distributed across all regions. The Ugandan government is encouraging public-private partnerships where private health facilities contribute to purchasing some of the equipment to serve the population. For example, most of the lower range pieces of equipment are private owned across the regions which is in line with government policy of public-private partnership where government is in support of private institutions providing care for the population [21]. It has been highlighted that radiology equipment are expensive and this puts large demands on budget for public the sector. This may be the reason for the low levels of equipment to population distribution in Uganda. Therefore, when government efforts get supplemented by private institutions, this skewed distribution may be addressed to some extent. However, this may not alleviate the burden of imaging costs on the public or accessibility since the public will have to pay for it. Radiology and imaging manpower is also linked to availability and utilization and this may very well reflect the availability of radiology and imaging specialists [22]. That would mean that there are disparities in imaging and human resource distribution, with disproportionally more human resource in the central region compared to the other regions which are mainly rural.

This study is one of few studies conducted in sub-Saharan Africa. It brings to light several issues that might potentially be applicable across different low-resource settings. The first issue relates to the equipment to population ratio which is below the WHO recommended ratio. Attaining the WHO recommended numbers may require deliberate policy formulation and planning, together with strengthening the public-private-partnerships. Allocation of more funding to radiology and imaging across the country is recommended. The second issue speaks to the skewed distribution of imaging equipment and services in the country. This distribution is perhaps a reflection of what could be happening in many other low-resourced countries. The factors responsible for these disparities need to be studied, enumerated and purposefully addressed. Governments may have to redirect health resources from urban to rural where most people can barely afford the cost of private imaging services.There is need to work on rural electrification and improve incomes of rural populations [22]. Therefore, despite the challenges with financing and mal-distribution of radiology equipment, some improvements are still needed. For example, there is need to find ways of how to effectively utilize the available small numbers of imaging equipment for impacting patient management and improve treatment outcomes. This calls for evidence-based planning, and purchasing of equipment. The disease burden within communities and at health facilities dictate on the type, level of technological sophistication and number of equipment to be purchased [14,15]. Use of evidence-based clinical imaging guidelines is also likely to improve appropriateness of imaging decisions and supposedly influencing management and treatment decisions, and hopefully impacting treatment outcomes.

The major limitation of this study was that it did not carry out an ultrasound equipment audit. It can be argued that inclusion of the ultrasound equipment audit would have added more rigor to the study and improved equipment to population ratios. In addition, the study would perhaps have benefited from close interaction with key stakeholders responsible for purchasing, financing and planning for this equipment through some key informant interviews. This could possibly have provided a richer holistic picture and explanation for some of what we found out in this audit. However, findings from this study still provide a solid foundation to inform policy on distribution, purchasing and financing of radiology equipment. The Ministry of Health and Finance will potentially find these findings useful. This being the first study auditing radiology equipment in Uganda, more and continued efforts in health systems research highlighting the role of diagnostic imaging are encouraged to inform policy. An audit of ultrasound equipment would also be a good point for further research owing to the fact that ultrasound equipment has been reported to be widely available in the country.

## Conclusion

Uganda is still below the WHO recommended equipment per population ratio and there is skewed distribution of the equipment across the country. Most radiology equipment was functional and the data related to equipment was well kept. More financing for radiology equipment is needed to reduce this gap in equipment distribution. It is also noted that there is an effective public private partnership in Uganda concerning radiology and this should continue to be supported and strengthened. This may support the privately owned radiology facilities help to reduce inequalities and skewed distribution of imaging services and enable access to more sophisticated imaging technologies. Government may then redirect health resources to rural areas, where there is less interest for the private imaging sector.

### What is known about this topic

- Healthcare technology, including diagnostic imaging is an essential component of the health care system and is important for achieving Universal Health Coverage
- There is limited robust country-level data for diagnostic imaging globally;
- Three countries in sub-Saharan Africa have done similar studies (South Africa, Tanzania and Zimbabwe).

### What this study adds

- It demonstrates that the data for radiology equipment in Uganda is well kept and is accessible;
- It highlights the fact that Uganda has not met the WHO recommended radiology equipment per population ratio;
- There is a positive public private partnership in Uganda concerning radiology which may not be intentional but could be used by the Ugandan government to reduce the inequalities in radiology services for the Ugandan population.
